# Film measurement and analytical approach for assessing treatment accuracy and latency in a magnetic resonance‐guided radiotherapy system

**DOI:** 10.1002/acm2.13915

**Published:** 2023-03-19

**Authors:** Hiroki Nakayama, Hiroyuki Okamoto, Satoshi Nakamura, Kotaro Iijima, Takahito Chiba, Mihiro Takemori, Tetsu Nakaichi, Shohei Mikasa, Kyohei Fujii, Tatsuya Sakasai, Junichi Kuwahara, Yuki Miura, Daisuke Fujiyama, Yuki Tsunoda, Takuma Hanzawa, Hiroshi Igaki, Weishan Chang

**Affiliations:** ^1^ Radiation Safety and Quality Assurance Division National Cancer Center Hospital Chuo‐ku Tokyo Japan; ^2^ Department of Radiological Sciences Graduate School of Human Health Sciences Tokyo Metropolitan University, Higashioku Arakawa‐ku Tokyo Japan; ^3^ Department of Radiation Sciences Komazawa University Setagaya‐ku Tokyo Japan; ^4^ Department of Radiological Technology National Cancer Center Hospital Chuo‐ku Tokyo Japan; ^5^ Department of Radiological Technology National Cancer Center Hospital East Kashiwa Chiba Japan; ^6^ Department of Radiation Oncology National Cancer Center Hospital Chuo‐ku Tokyo Japan

**Keywords:** gating method, latency, magnetic resonance‐guided radiotherapy, quality assurance

## Abstract

**Purpose:**

We measure the dose distribution of gated delivery for different target motions and estimate the gating latency in a magnetic resonance‐guided radiotherapy (MRgRT) system.

**Method:**

The dose distribution accuracy of the gated MRgRT system (MRIdian, Viewray) was investigated using an in‐house‐developed phantom that was compatible with the magnetic field and gating method. This phantom contains a simulated tumor and a radiochromic film (EBT3, Ashland, Inc.). To investigate the effect of the number of beam switching and target velocity on the dose distribution, two types of target motions were applied. One is that the target was periodically moved at a constant velocity of 5 mm/s with different pause times (0, 1, 3, 10, and 20 s) between the motions. During different pause times, different numbers of beams were switched on/off. The other one is that the target was moved at velocities of 3, 5, 8, and 10 mm/s without any pause (i.e., continuous motion). The gated method was applied to these motions at MRIdian, and the dose distributions in each condition were measured using films. To investigate the relation between target motion and dose distribution in the gating method, we compared the results of the gamma analysis of the calculated and measured dose distributions. Moreover, we analytically estimated the gating latencies from the dose distributions measured using films and the gamma analysis results.

**Results:**

The gamma pass rate linearly decreased with increasing beam switching and target velocity. The overall gating latencies of beam‐hold and beam‐on were 0.51 ± 0.17 and 0.35 ± 0.05 s, respectively.

**Conclusions:**

Film measurements highlighted the factors affecting the treatment accuracy of the gated MRgRT system. Our analytical approach, employing gamma analysis on films, can be used to estimate the overall latency of the gated MRgRT system.

## 1 INTRODUCTION

Tumor motion should be properly managed to ensure the accurate delivery of a prescribed dose. If a tumor moves significantly during dose delivery, some doses can be inaccurately delivered to the tumor and surrounding normal tissues, potentially causing local control failure and radiation toxicity in the worst‐case scenario. Various factors, such as organ peristalsis, body motion, and respiration, can affect tumor mobility. Respiratory motion increases the uncertainty associated with the treatment accuracy in radiotherapy processes, such as computed tomography (CT) scans,^1^, ^2^, ^3^ treatment plans associated with dose calculation,^4^ and treatment‐related dose delivery involving interplay effects.^5^

To compensate for tumor motion, several clinicians apply the treatment‐region standard presented in the International Commission on Radiation Units and Measurements (ICRU) 62.^6^ Based on the ICRU report, the internal target volume (ITV) can be determined to compensate for tumor motion as well as the variations in size, shape, and position of a tumor during treatment. In addition, the ITV margin comprises inter‐ and intra‐fractional variations that account for tumor motions between daily treatments and tumor motions during a single treatment, respectively. The intrafractional variations can be compensated for using the following techniques: (1) motion‐encompassing,^7^, ^8^, ^9^ (2) breath‐holding,^10^, ^11^, ^12^ (3) forced shallow breathing,^13^ (4) real‐time tumor tracking,^14^, ^15^, ^16^ and (5) respiratory‐gating methods.^17^, ^18^, ^19^ In clinical practice, the respiratory‐gating method commonly employs a dedicated device, such as the Real‐time Position Management system (Varian Medical Systems, Palo Alto, CA, USA), which is positioned on a patient's abdomen or chest. Besides, many clinicians employ stereotactic kilovoltage x‐ray imaging systems, which detect fiducial markers as surrogate signals of tumor mobility.^19^ Unwanted dose delivery to normal tissues surrounding a tumor can be reduced using a narrow gating window. However, the usage of a narrow gating window decreases the fraction of active time of a radiation beam with respect to the total treatment time (i.e., the duty cycle). Increasing the treatment time increases the likelihood of unexpected organ motion that results in the movement of the tumor and organs to an undesired position.^20^ The American Association of Physicists in Medicine Task Group 76 has described each compensating approach in detail.^21^

Our institution has clinically implemented MRIdian (Viewray, Oakwood Village, OH, USA)—a radiation treatment machine with an integrated magnetic resonance imaging (MRI) system and a treatment system with three ^60^Co sources. MRIdian offers fixed gantry step‐and‐shoot intensity‐modulated radiotherapy (IMRT), and the gating method using cine MRI. Cine MRI in MRIdian can monitor intrafractional tumor motions at a time resolution of 0.25 s. Other clinical gating implementations use a gating level and window based on respiratory motion, whereas MRIdian defines a boundary with an extended margin for tracking a target on cine MRI. A tracking target is usually a tumor, but other organs showing the tumor motion can also be tracked when the tumor is invisible on cine MRI. Before initiating treatment, an operator delineates the tracking target. During the treatment, continuous delineations of the tracking target on cine MRI are automatically generated. MRIdian performs the gating method in two beam states: “beam‐on” and “beam‐hold”. The “beam‐on” state means that the beam is delivered when a fraction of the tracking target is within the boundary. The fraction is decided by a clinician in advance. The “beam‐hold” state delays the treatment (stops the beam) when the fraction of the tracking target is outside the boundary. Lamb et al.^22^ investigated dose distribution using MRIdian using a phantom that moved synchronously with the respiratory waves of real patients and artificial waves. They demonstrated that dose distribution and the passing rate in a gamma analysis vary with respect to the target motion (different duty cycles and velocities). However, the extent to which dose distribution depends on the number of beam switches and tumor velocity has not been clearly demonstrated for the gating method. Moreover, various studies have been conducted using different approaches to quantify the inherent latency of the gating method via a conventional linear accelerator (LINAC) system.^23^, ^24^, ^25^, ^26^ Latency measurements of the gating method in MRIdian must show that the performance meets the recommendation by Task Group 76, which stipulates a latency of <0.5 s in tracking/compensation.^21^ The overall latency of magnetic resonance‐guided radiotherapy (MRgRT) systems, particularly MRIdian, is caused by three processes: MR imaging, gating‐signal processing, and radiation‐source movement. Borman et al.^27^ investigated latency during the acquisition of magnetic resonance (MR) images. Green et al.^28^ described an acceptance test along with commissioning, quality assurance (QA), and flow procedures when using MRIdian and measured the overall latency of the gating method using an oscilloscope.

In this study, we investigate the effect of the number of beam switches, target velocity, and gating latency on the dose distribution and estimate the overall latency of beam switches in gated MRgRT. For these purposes, we propose a novel analytical approach using a film and an in‐house‐developed MR‐compatible phantom for MRgRT.

## 2 MATERIALS AND METHODS

### 2.1 In‐house‐developed phantom

**FIGURE 1:**
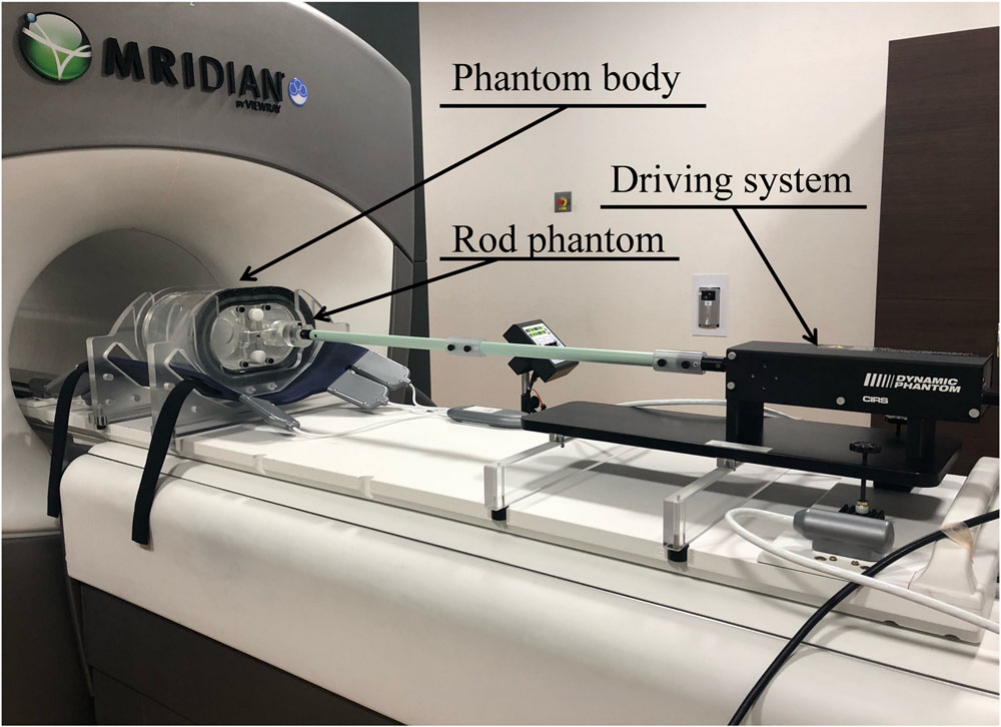
External appearance of the newly developed phantom. Shown are the phantom body, the rod phantom inserted into the phantom body, and a driving system (Viewray Dynamic Phantom). The phantom body comprises a water‐fillable acrylic shell and driving system attached to the rod phantom

**FIGURE 2:**
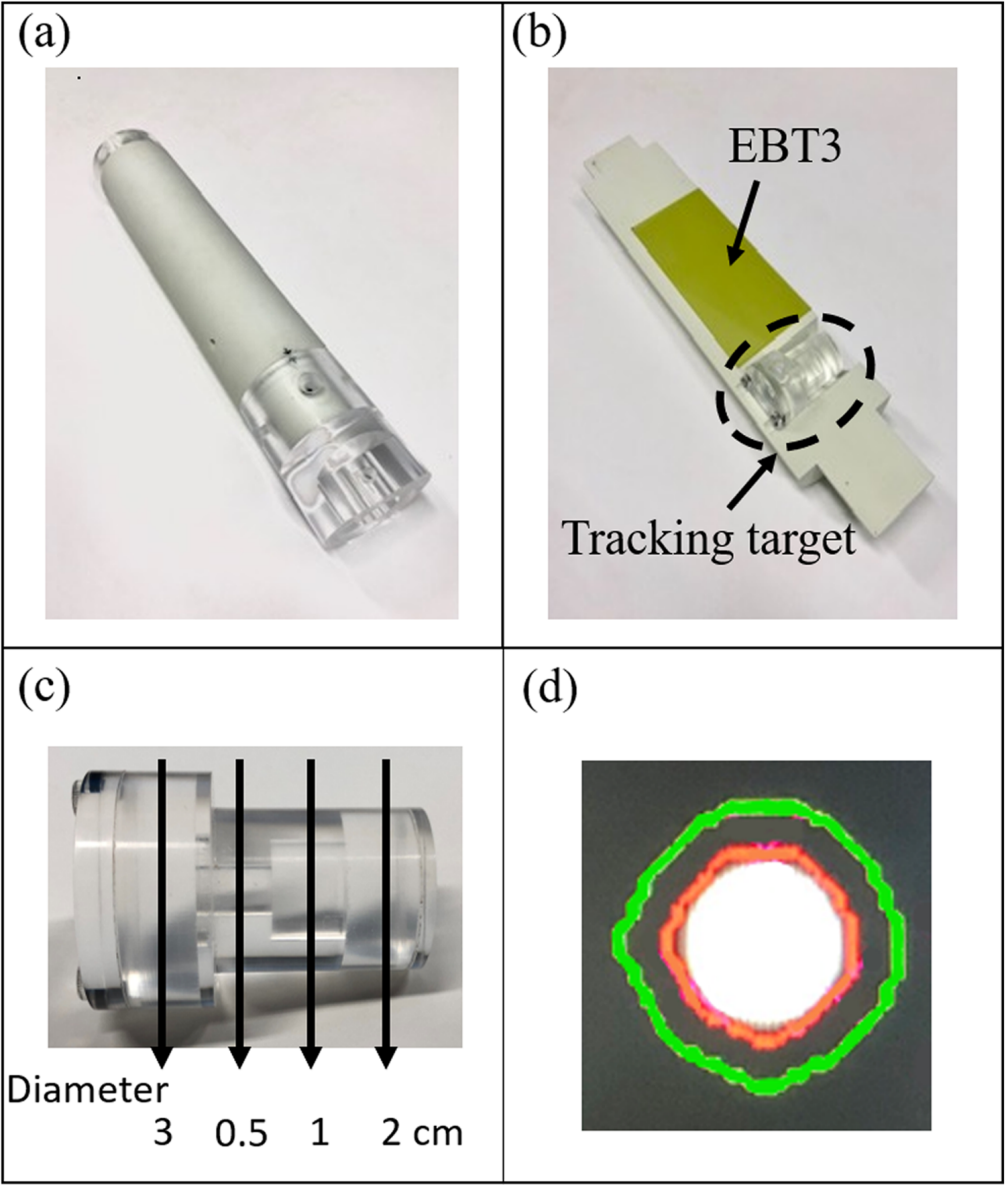
In‐house developed rod phantom: (a) picture of the rod phantom. (b) Inner view of the rod phantom; the two spaces inside the rod phantom accommodating the simulated target (c), and the film is designed to measure the dose distributions. (c) Simulated target containing four cylinders with different diameters (0.5, 1, 2, and 3 cm). (d) Example of target tracking in cine MRI. The white circle is the tracked target, the exterior red line shows the autocontouring of the tracking target by the MRIdian system, and the outermost green line is the boundary (at 3 mm from the tracking target) that triggers switching between beam‐hold and ‐on

Our in‐house‐developed phantom comprised a phantom body, a rod phantom, and a driving system (Figure 1). The phantom body was constructed from an acrylic shell that could be filled with water. A simulated target and a film were placed inside the rod phantom (Figure 2a,b) to obtain the dose‐distribution measurements collected in the gating method. The rod phantom was made of a water‐equivalent material (plastic water, CIRS, Inc., Norfolk, VA, USA). The physical and electron densities of plastic water were 1.039 g/cm^3^ and 3.345 × 10^23^/cm^3^, respectively.^29^

The simulated target in the rod phantom (Figure 2c) was constructed using a water‐fillable acrylic shell. Because the simulated target could be placed in the pocket of the rod phantom without requiring adhesive, another simulated target with a different shape could be installed in the same rod phantom. This simulated target was designed to be detected only in the sagittal plane of cine MRI. Four cylinders with different diameters (0.5, 1, 2, and 3 cm, indicated by arrows in Figure 2c) were considered for the second simulated target and imaged as circular targets in various sagittal planes of cine MRI. Figure 2d depicts a representative cine MRI image of a simulated circular target. As the simulated target is a water‐fillable acrylic shell, its signal can be changed by adding a contrast agent. Accordingly, the in‐house‐developed phantom allows the measurement of simulated targets with various shapes, sizes, and signals. In this study, the signals of the circular tracking targets were enhanced using a water‐soluble contrast agent. The water‐to‐contrast‐agent ratio was set to 1:0.01, at which the signal was the strongest in our previous measurements.

A driving system for motion phantoms (Viewray Dynamic Phantom, Model: 008 V Dynamic Phantom, CIRS Inc., Norfolk, VA, USA) was attached to the above‐described rod phantom and connected to a control personal computer (PC) outside the treatment room. Then, arbitrary motion patterns of the rod phantom and simulated targets were imported to the PC.

### 2.2 Treatment planning

**FIGURE 3:**
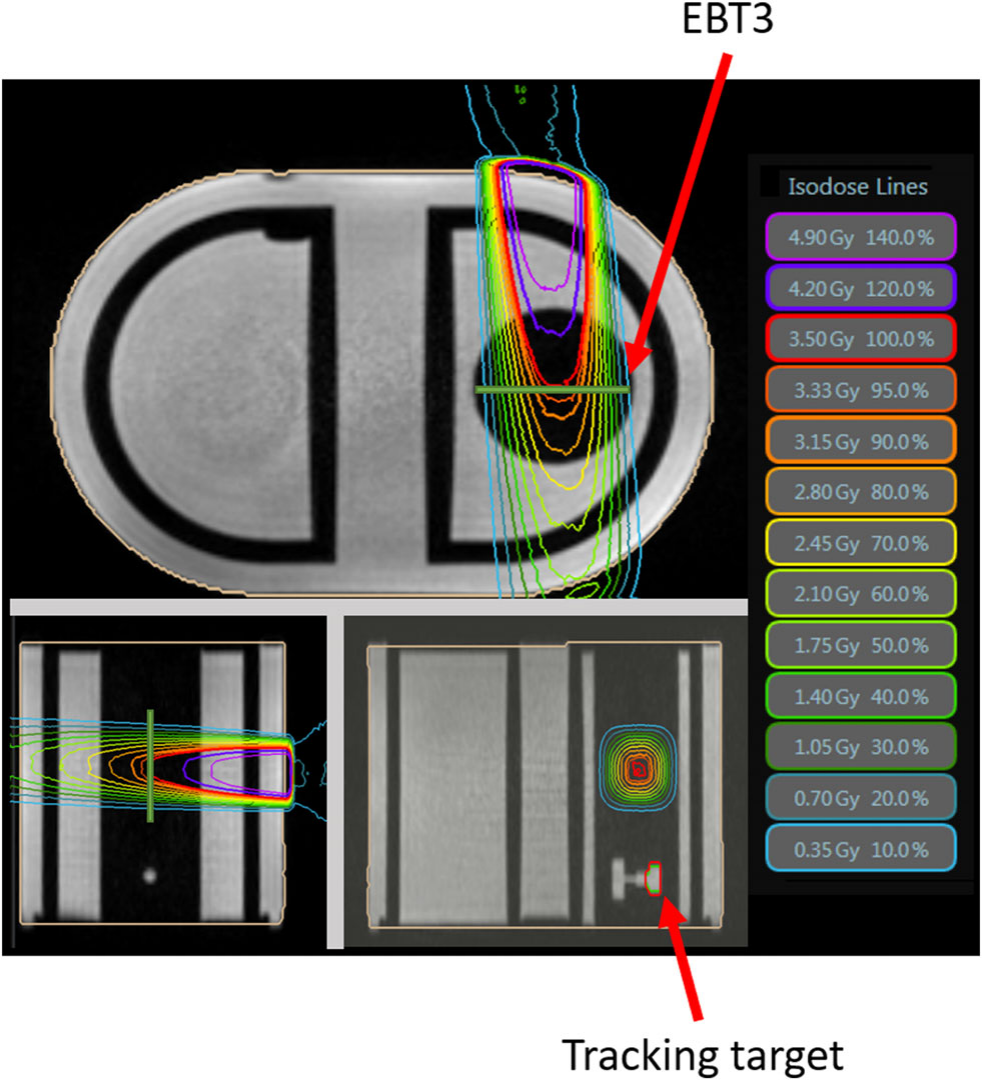
Example of the treatment plan. White parts are the water‐filled areas, and the black parts are the acrylic shell and rod phantom. In this treatment plan, the field size is 4.2 × 4.2 cm^2^ and the prescribed dose to the film is 3.5 Gy

MR images of the in‐house‐developed phantom for a treatment planning were obtained as an image sequence via true fast imaging with steady‐state‐free precession (TRUFI)^30^ in MRIdian. The field of view (FOV) and resolution of the images were 40 cm × 40 cm × 43 cm (left–right [LR] × anterior–posterior [AP] × superior–inferior [SI]) and 0.15 cm (LR, AP) × 0.30 cm (SI), respectively. The TRUFI sequence was also used in cine MRI. CT images (Aquilion ONE NATURE Edition, Canon Medical Systems, Tokyo, Japan) of the in‐house‐developed phantom with a matrix size of 512 × 512 and a slice thickness of 3 mm were acquired to obtain an electron density map for calculating the dose distribution. The MR and CT images were transferred to the Viewray treatment planning system (TPS). Following the image registration between the MR and CT images, the treatment plan was designed with a field size of 4.2 × 4.2 cm^2^ at the isocenter. The prescribed dose delivered to the film was 3.5 Gy, and 358.6 ± 1.43 s was required to deliver the dose. The dose calculation was performed to account for the influence of the magnetic field (*B*_0_). The dose distribution of the treatment plan is shown in Figure 3.

### 2.3 Gating method

#### 2.3.1 Setting of radiotherapy

The treatment couch position where the phantom was on was adjusted if necessary, and the phantom's position was decided based on the results of an image‐guided technique. Afterward, the rod phantom's origin was determined from a marker line drawn on it. The FOV and resolution of the setup image were 40 cm × 40 cm × 43 cm (LR × AP × SI) and 0.15 cm × 0.15 cm (LR, AP × SI), respectively. For precise detection of the tracking target on cine MRI, the MRI parameters for the setup differ slightly from those of the treatment planning images. By selecting a proper sagittal plane, the simulated circular target with a diameter of 2 cm was used as the tracking target in the gating method. The contour of the tracking target was delineated by a signal threshold (minimum: 0; maximum: 280) to reduce interobserver delineation errors. The FOV and resolution of cine MRI were 35 cm × 35 cm (AP × SI) and 0.35 cm × 0.5 cm (AP, SI × LR), respectively, and the boundary was created by expanding the tracking target by 3 mm. The allowed excursion in the functional setting of cine MRI was 0%; thus, the beam‐on and ‐hold functions became active just when the tracking target reached the boundary.

#### 2.3.2 Target motion

**FIGURE 4:**
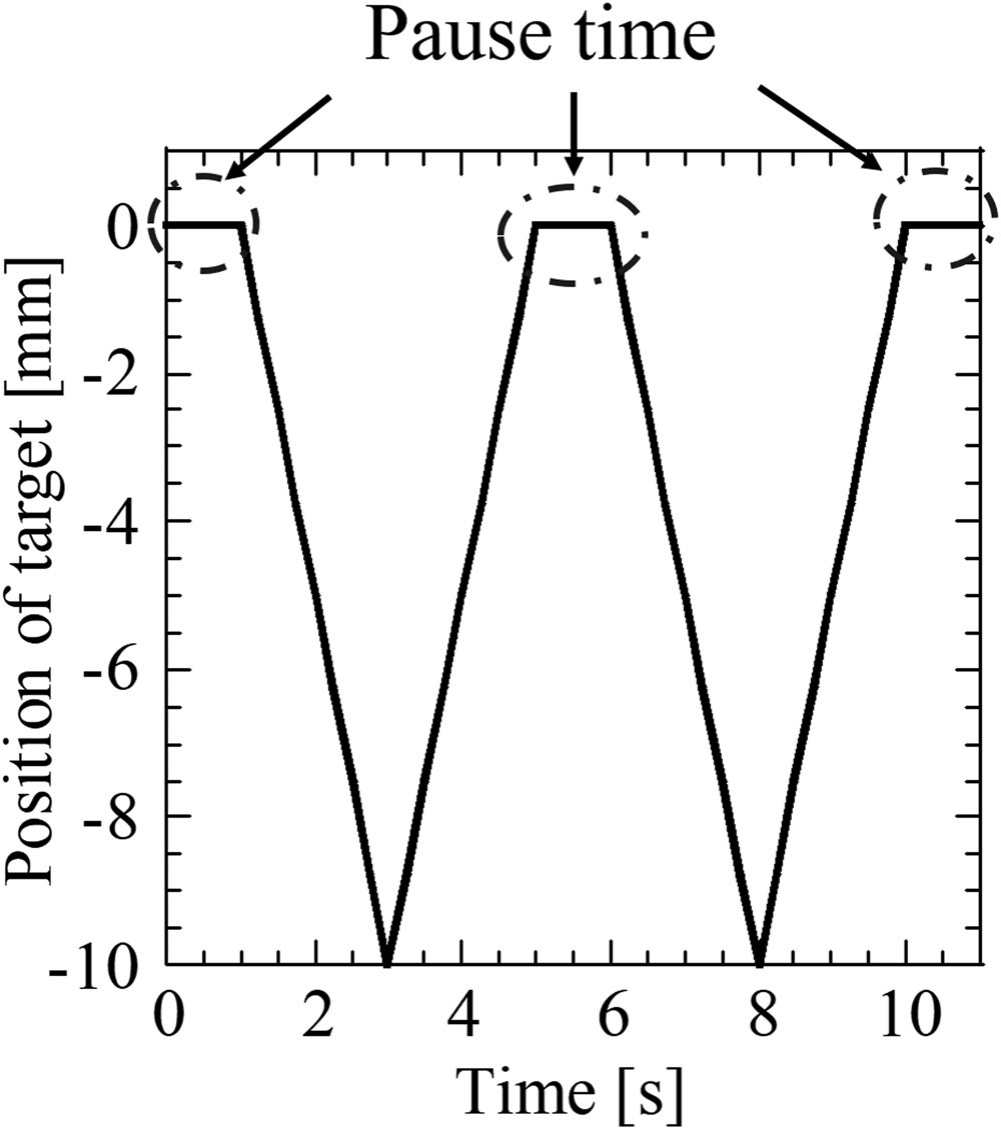
Example of a sawtooth wave applied to the target motion as the phantom was moved in the superior–inferior (SI) direction. The origin of the phantom's position is at 0 mm on the vertical axis and the lowest position is −10 mm, meaning that the phantom moved by 10 mm in the inferior direction. The pause time defines the interval between periodic motions of the target

To observe the effects of target motion on the delivered dose distribution, various sawtooth waves as the motion of the in‐house‐developed phantom were imported to the control PC. Figure 4 shows an example of the target motion in the SI direction, where the inferior direction lies along the negative perpendicular axis. According to the control PC, the in‐house‐developed phantom moved by 10 mm in the inferior direction. To observe the effect of the duty cycle and the number of beam switches on dose distribution, we periodically moved the target at a constant velocity of 5 mm/s while varying the pause time between the motions (0, 1, 3, 10, and 20 s). A velocity of 5 mm/s was determined by a preliminary examination on the tumor motions of cine MRI for 20 patients with pancreatic cancer treated in our institution. The average tumor velocity of the 20 patients, determined using cine MRI, was 5.1 ± 2.1 and 6.1 ± 2.4 mm/s during inspiration and expiration, respectively. As pancreatic cancer is among the most MRIdian‐treated cancers in our institution, the average velocity of pancreatic tumors was applied in this study. Moreover, dose distributions were measured in phantoms moving with different velocities (3, 5, 8, and 10 mm/s) without any pause time (pause time = 0 s) and in a stationary phantom.

### 2.4 Film measurements and analysis

Dose distributions were measured on radiochromic films (EBT3, Ashland Inc., Bridgewater, NJ) from a single production lot (lot number: 04022004). The films were attached to the rod phantom and marked with two horizontal and two vertical dots. The intersection of the two lines connecting opposite pairs of the dots represented the center of the radiation field. The change in dose distribution was measured based on this coordinate. The films were scanned 24 h after irradiation using a flatbed scanner (ES‐G11000, EPSON, Nagano, Japan) with a resolution of 72 dpi. To establish a calibration curve, one film was irradiated with different doses (40–450 cGy) using 6‐MV x‐rays from a LINAC (TrueBeam, Varian Medical Systems, Palo Alto, CA, USA). The measured and planned dose distributions were compared by gamma analysis to evaluate the effects of varying target motions. Each dose distribution was normalized to 100% at the maximum dose. In the gamma analysis, the minimum dose threshold was 30% of the prescribed dose and the strict passing criteria of 3% and 1 mm were applied to identify the difference in dose distribution because we wanted to make the difference noticeable. Four or more measurements were performed for each motion to investigate the reproducibility of the analysis.

### 2.5 Latency evaluation

The overall latency was estimated from the measured dose distributions on the films using an analytical MATLAB program (ver. R2019b, The MathWorks, Inc., Natick, MA, USA). First, the dose distribution of the treatment plan for a stationary target was divided by the irradiation time calculated in the treatment plan to obtain the dose distribution per unit time (hereinafter, the dose distribution rate). This dose distribution rate was shifted following the target motion as specified in C.2, that is, the analytical program was run under the irradiation conditions as the film measurements. The target motions used in the program were those with the target velocities of 3, 5, 8, and 10 mm/s without pause time because when the number of beam switches is large, the component of dose distribution shifts resulting from the latency would be dominant; consequently, the latency can be properly estimated. In this program, two latencies corresponding to beam‐hold and beam‐on were investigated separately as variable parameters. The dose distribution rate was calculated based on the following conditions.

1. Latency of beam‐hold—the target is over irradiated when it moves beyond the boundary. In the SI coordinates, the calculation range of the dose distribution rate is as follows:
$$R_{\mathrm{Hold},i}\text{: from 0 to}\;-3\;\text{mm}\;\left(\mathrm{boundary}\right)-V_{t}\times T_{\text{Lat-Hold},i}$$
2. Latency of beam‐on—the target is under irradiated when it moves inside the boundary. The sampling range is as follows:
$$R_{\mathrm{On},j}\text{: from 0 to}-3\;\text{mm}\;\left(\mathrm{boundary}\right)+V_{t}\times T_{\text{Lat-On},j}$$

**FIGURE 5:**
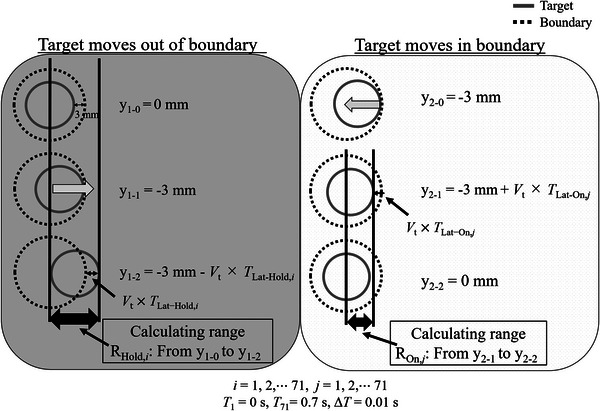
Ranges of calculated dose distributions of a moving target at beam‐hold, where the target moves outside the boundary, and beam‐on, where the target moves within the boundary. y_1‐0_: origin of motion (0 mm); y_1‐1_: coordinate at which the target reaches the boundary (−3 mm); y_1‐2_ coordinate of beam switching after the latency of beam‐hold passing; y_2‐0_: coordinate at which the target touches the boundary (−3 mm); y_2‐1_: coordinate of beam‐on switching after the latency of beam‐on passing; y_2‐2_: origin of motion (0 mm). *T*_Lat‐Hold,_ *_i_* and *T*_Lat‐On,_ *_j_* denote the latencies of beam‐hold and beam‐on, respectively. Each latency was varied from 0 to 0.7 s at 0.01 sintervals(i=1,2,⋯71,j=1,2,⋯71). *V*_t_ is the velocity of the target motion. R_Hold,_ *_i_* and *R*_On_ *_,j_* are the calculation ranges of the target movement, which depend on the latency input to the analytical program

**FIGURE 6:**
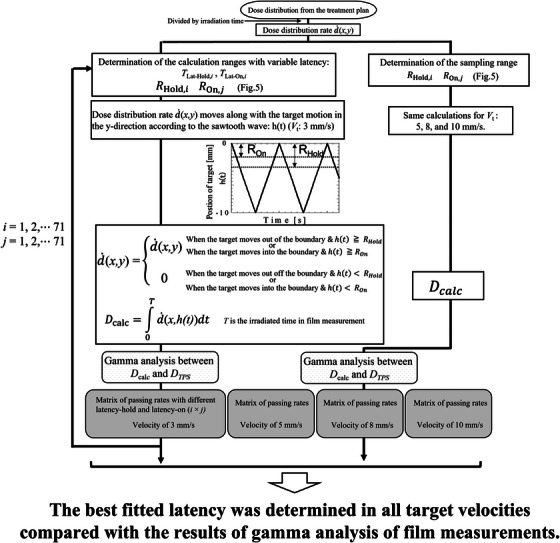
Flowchart of the analytical approach for estimating the overall latency. The planned dose distribution was divided by the irradiation time at TPS to obtain the dose distribution per unit time (the dose distribution rate $\dot{d}$(*x,y*)), which moves along the target motion (*V*_t_) at the specified velocity (3, 5, 8, or 10 mm/s) with no pause time (recreating the motion of the target during film measurements). The dose distribution rates were calculated within the ranges *R*_Hold,_ *_i_* and *R*_On,_ *_j_* (see Figure 5). Outside of these ranges, the dose distribution rates were regarded as those at beam‐hold time, were not calculated. *T*_Lat‐Hold,_ *_i_* and *T*_Lat‐On,_ *_j_* are the latencies of beam‐hold and beam‐on, respectively, which are input separately as variable parameters in the analytical program (0 to 0.7 s at 0.01 sintervals)(I=1,2,⋯71,j=1,2,⋯71). The sampled dose distribution rates were accumulated over the time of film measurements and the dose distribution *D*_calc_ calculated by the program replicated the dose distribution by gated radiotherapy with a moving target. The dose distributions from the TPS (*D*_TPS_) were compared with *D*_calc_ in the gamma analysis. These results of gamma analysis from *D*_calc_ and *D*_TPS_ and from film measurements and *D*_TPS_ were compared and we defined a pair of input latency as the overall latency when the difference of the results of gamma analysis mentioned above was the smallest

*R*_Hold,_ *_i_* and *R*_On_ *_,j_* denote the calculation ranges when the target moves outside and inside the boundary, respectively. These parameters depend on the latency input to the analytical program. *V*_t_ denotes the target motion velocity, and *T*_Lat‐Hold,_ *_i_* and *T*_Lat‐On,_ *_j_* denote the latencies during beam‐hold and beam‐on, respectively. The input latencies at the beam‐hold and beam‐on were independently varied from 0 to 0.7 s at 0.01 s intervals (i=1,2,⋯71,j=1,2,⋯71). This analysis ignored the dose distribution rate outside these two ranges (*R*_Hold,_ *_i,_ R*_On_ *_,j_*) because MRIdain did not irradiate on out‐of‐range areas. The above‐described ranges are shown in Figure 5. The dose distribution rates during the irradiation time of the films were summed based on the target motion read in the analytical program. These calculations were performed at each target velocity. The method for estimating the overall latency is described below. One pair latency is input in the analytical program and one dose distribution is made at each target velocity (3, 5, 8, and 10 mm/s), as a dose distribution is calculated at each target velocity. The dose distribution calculated in the analytical program is compared with that calculated in TPS using gamma analysis (the criteria of gamma analysis were the same as those in film measurements); then, four gamma pass rates are calculated in all velocities. The gamma pass rates mentioned above and the average gamma pass rate calculated between the measured dose distribution and the one in TPS (according to Figure 11) are compared at each velocity. The differences in gamma pass rates in all velocities were summed, and the residual errors are obtained at all velocities at the pair of input latency. As the number of input latency was 5041 (because the input latencies at beam‐hold and beam‐on were independently varied from 0 to 0.7 s at 0.01 s intervals); thus, the number of residual errors is also 5041. We defined a pair of input latency as the overall latency when the residual error was the smallest. The flowchart of the analytical program is shown in Figure 6.

## 3 RESULTS

### 3.1 Film measurement

**FIGURE 7:**
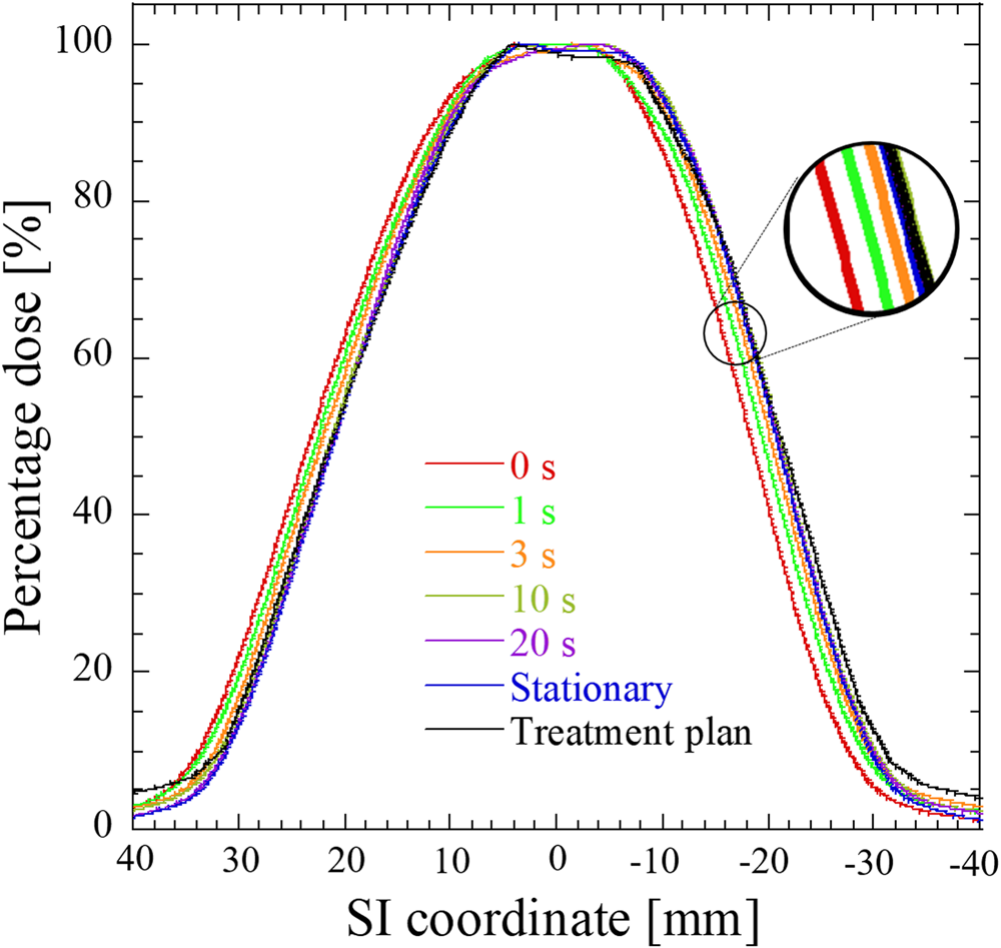
Representative dose distributions in the treatment plan and in experiments with stationary and moving targets. The values 0, 1, 3, 10, and 20 s are the pause times for the moving target. Along the horizontal axis, the positive and negative coordinates denote superior and inferior positions relative to the origin of the target position, respectively. Each dose distribution is normalized to 100% of the maximum dose

**FIGURE 8:**
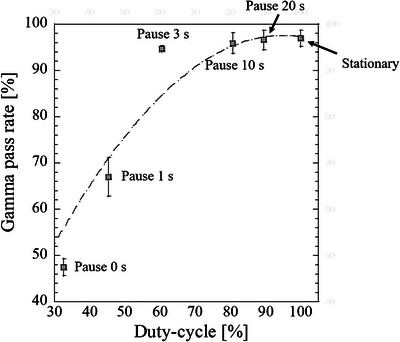
Relationship between duty cycle and gamma pass rates in each target motion, referenced to the dose distribution of the treatment planning. The duty cycle denotes the ratio of beam‐on time to the total treatment time. Error bars depict the standard errors (SEs)

**FIGURE 9:**
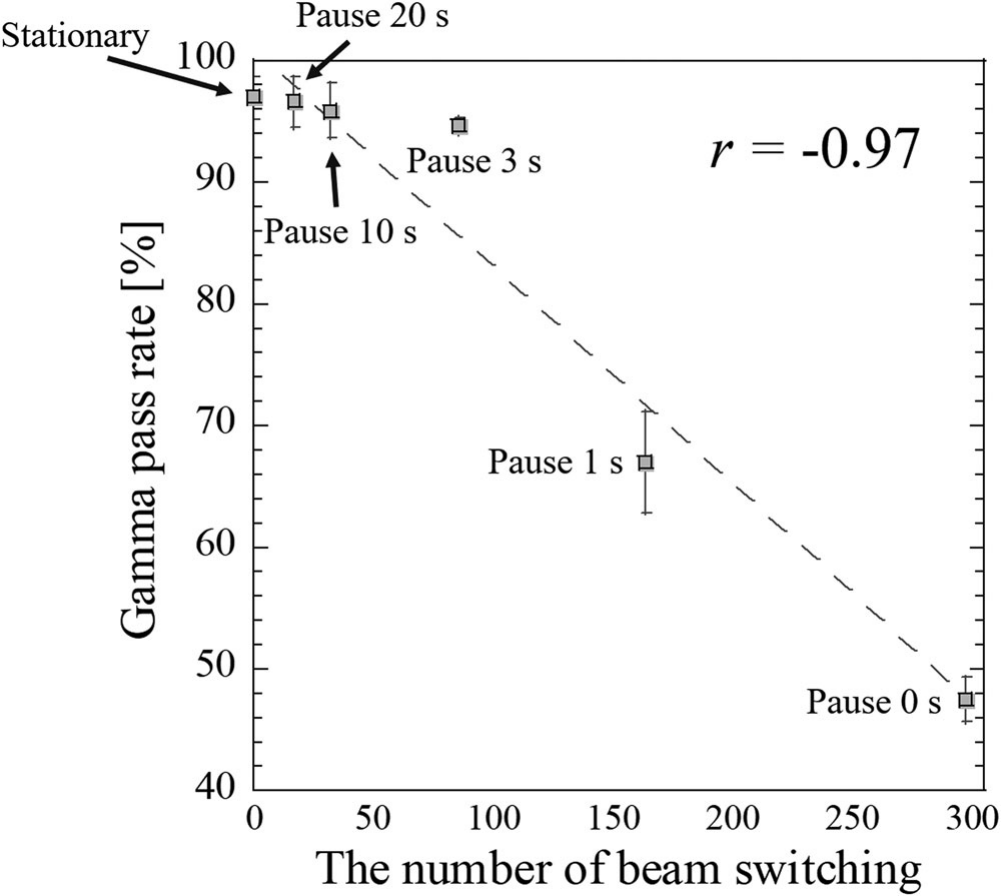
Relationship between the number of beam switches (beam‐hold and beam‐on) and gamma pass rate for different target motions, referenced to the dose distribution in the treatment planning. r is the Pearson correlation coefficient between the number of beam switches and the gamma pass rate. Error bars depict the SEs

**FIGURE 10:**
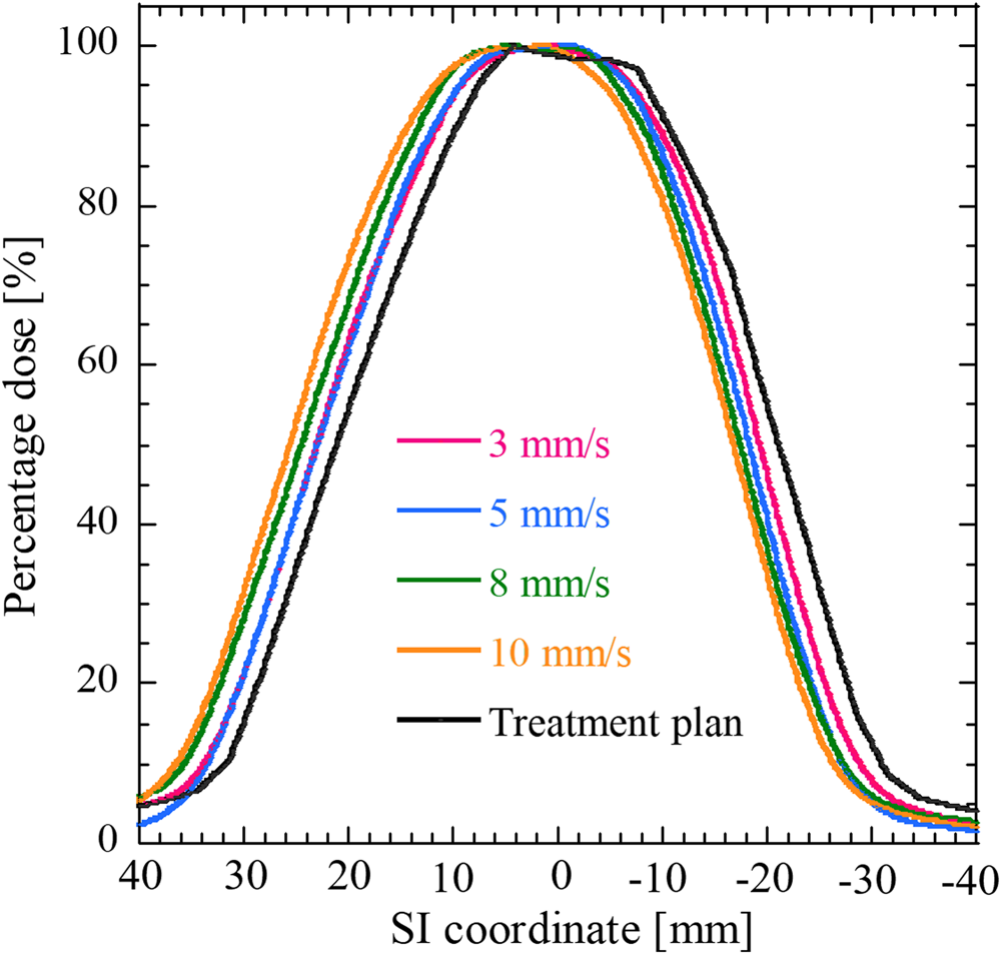
Representative dose distributions of targets moving at different velocities. Along the horizontal axis, the positive and negative coordinates denote superior and inferior positions relative to the origin of the target position, respectively. Each dose distribution was normalized to 100% of the maximum dose

**FIGURE 11:**
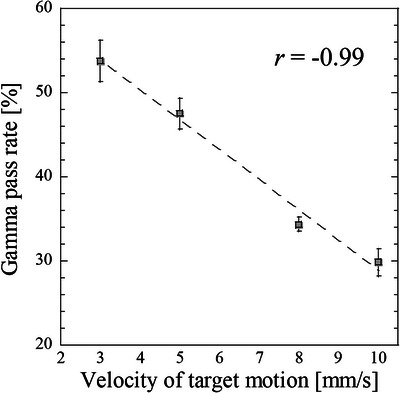
Relationship between target velocity and gamma pass rate, referenced to the dose distribution from the treatment planning. Error bars depict the SEs

Figure 7 displays sample dose profiles of the stationary and moving films at each pause time along the SI direction as well as a TPS dose profile. Shortening the pause time (i.e., reducing the duty cycle) increased the dose profile shift. Figure 8 shows the relationship between the duty cycle and gamma pass rates, and Figure 9 shows the relationship between the gamma pass rates and number of beam switches required to deliver the prescribed dose (3.5 Gy) to the film. One switching beam included one beam‐hold function and one beam‐on function. The gamma pass rate strongly correlated with the number of beam switches (Pearson's correlation coefficient *r* = −0.97). Further, the sample dose profiles along the SI direction were collected at various target velocities (3, 5, 8, and 10 mm/s) with no pause time. The results, along with a TPS dose profile, are shown in Figure 10. Increasing the target velocity induced larger dose profile shifts. As shown in Figure 11, target velocity strongly correlated with gamma pass rate (Pearson's correlation coefficient *r* = −0.99).

### 3.2 Latency measurement

The overall latencies of beam‐hold (0.51 s) and beam‐on (0.35 s) were derived based on the film measurements performed using the analytical program. The uncertainties associated with the estimated latencies are listed in Table 1. The standard uncertainties associated with the latencies (type A) of beam‐hold and beam‐on were ± 0.13 and ± 0.02 s, respectively. Other factors contributing to the uncertainties were the accuracy of the moving phantom, the setup accuracy of the image‐guided radiation therapy (IGRT) at MRIdian, and the positional accuracy of the marker dots drawn on the films. The motion accuracy (standardized by the CIRS company) was ± 0.1 mm. The accuracy of IGRT at MRIdian was estimated using our daily QA measures; the median and standard deviation were 0.0 and 0.3 mm, respectively. The positional accuracy of the marker on the films was determined from the scanned image resolution of the films. Owing to the scanning rate of the irradiated films (72 dpi), the positional accuracy of the irradiated films was approximately 0.35 mm/pixel. The combined standard uncertainties of the latencies with a coverage factor of 1 were estimated to be 0.17 and 0.05 s for beam‐hold and beam‐on, respectively.

**TABLE 1:** Uncertainty for latency estimation

Source of uncertainty	Value	Uncertainly of the latency at beam‐hold [s]	Uncertainly of the latency at beam‐on [s]
Type A (film measurement)		0.13	0.02
Type B			
Motion accuracy of motion phantom	±0.1 mm	0.03	0.02
Set‐up accuracy by IGRT	±0.3 mm	0.06	0.02
Readout of the makers on film	±0.35 mm	0.09	0.03
Combined uncertainty (*k* = 1)		0.17	0.05

*Note*: *k* is a coverage factor.

## 4 DISCUSSION

In this study, we investigated how the number of beam switches and target motion velocity influenced the dose distribution when using gating methods in MRIdian. In addition, a novel analytical approach for estimating the gating latency in MRIdian was proposed.

In most previous studies, the effects of tumor motion on MRI or dose distribution were evaluated using the QUASAR MRI‐4D motion phantom, QUASAR respiratory motion phantom (Modus Medical Devices Inc., London, Canada),^31^, ^32^, ^33^, ^34^, ^35^, ^36^, ^37^, ^38^ MRgRT motion management QA phantom, CIRS thorax phantom (CIRS, Inc., Norfolk, VA, USA),^22^, ^36^, ^39^, ^40^ and phantoms fabricated by authors.^41^, ^42^, ^43^, ^44^ In addition to these, some scholars used in‐house‐developed targets driven by a readymade driving system.^22^, ^34^, ^35^, ^38^, ^40^ The phantom developed by Lamb et al. contains custom rods filled with three high‐contrast materials and enables the attachment of films for the dosimetry studies.^22^ Meanwhile, our in‐house‐developed phantom allows different concentrations of the injected water‐mixed contrast agent, thereby obtaining variable MRI tracking target signals. This phantom can simulate real situations in which different patients show different contrasts between the tumor and normal tissues. In this study, simulated target signals were enhanced using the contrast agent to estimate the gating latency in the absence of factors that can influence the latency estimates. In the tests without the contrast agent, beam switching was observed when the actual target did not reach the boundary. This was attributed to the autocontouring distortion of the tracking target. The analytical program assumes a fixed distance of 3 mm between the position of the original target and the boundary. Consequently, the analytical program may calculate incorrect latency if the films are irradiated via autocontouring with some distortion. Because the contrast agent suppressed this distortion, it was used in this study. Additionally, simulated targets of various shapes can be selected to measure the tracking precision of the gating method under various situations. Herein, only a circular target was evaluated. In the future, the precision of autocontouring and gating within the gated MRgRT will be evaluated using different intensity signals and shapes of the tracking targets.

After decreasing the duty cycle or increasing the target velocity, significant differences appeared between the measured dose distributions of the moving and stationary targets (Figures 7 and 10; also observable in the TPS dose profiles). These differences can be explained by the absence and presence of latency. In the absence of latency, the boundary triggering the beam switching in the gating method was set to 3 mm from the tracking target. Because the tumor was irradiated until it crossed the boundary, increasing the time for which the tracking target remained within the boundary and not at original position of target increased the dose‐distribution shift. These effects may become more profound when a large dose is prescribed or the number of respiratory events increases. From a latency perspective, these effects are related to the overall latency of gating for the specific treatment machine. The effect of latency on dose distribution became significant when the number of beam switches is large. Moreover, the effect of target motion velocity on dose distribution is enhanced at higher velocities because the target can move more freely until the beam switching is performed. Gamma pass rate is strongly correlated with the number of beam switches (Figure 9). The gamma pass rate reportedly decreases at small duty cycles,^22^ but this study suggests that discrepant dose distributions can result from different numbers of beam switches. The same duty cycle can accommodate different numbers of beam switches and (according to our study) potentially allow different dose distributions.

In this study, the latencies of beam‐hold and beam‐on were determined as 0.51 ± 0.17 and 0.35 ± 0.05 s, respectively, consistent with those of Green et al.^28^ (0.246–0.527 s; average 0.394 s) and Lamb et al.^22^ (0.436 s). Our methodology for estimating latency based on film measurements and an analytical approach can evaluate the total effects of latency without dealing with the electrical signals of detectors. In this sense, our method differs from conventional methods.

To minimize the effects of target motion on dose distribution in clinical situations, the planning target volume (PTV) and boundary margins should be properly selected considering the target motions. For instance, a target traveling at 5 mm/s across a 3 mm boundary moves by approximately 5.5 mm before beam‐hold (the 3 mm distance to the boundary + 2.5 mm during the latency period of 0.5 s). If the PTV margin is 5 mm, part of the target is not irradiated immediately before beam‐hold. Expanding the PTV margin will eliminate this effect but risks the irradiation of unwanted doses to the surrounding normal tissues. Another strategy involves maintaining a small boundary margin, which allows prompt beam switching by the system functions but worsens the duty cycle. In this study, we recommend a breath‐hold technique that minimizes the effect of target motion in gated radiotherapy.

We used a single field to investigate the dosimetric influence of motion alone. The resulting dose distribution shifts cannot be attributed to the uncertainties associated with the positional accuracy of multi leaf collimator and gantry rotation. Although the dose distributions in IMRT differed from those of a simple field, our results are useful for evaluating the dosimetric impacts on IMRT dose distributions because the same shifts in dose distributions are expected in each segmented field of IMRT.

Nevertheless, this study has some limitations. First, our latency estimation ignores the shutter effect due to the movements of ^60^Co sources, that is, the dose distribution from the TPS divided by the treatment time in the analytical program for estimating the overall latency was assumed constant. However, the dosimetric discrepancy due to the shutter effect may be small because the source moved at high speeds. Second, as the gamma analysis based on film measurements was two‐dimensional, the effect of gating latency on three‐dimensional dose distributions remains uncertain. Third, the waveforms of tumors produced by the respiratory systems of patients might differ from the sawtooth waves analyzed in this study. Fourth, the analyzed tracking target had a simple shape, and it was highly contrasted against the surrounding material. In future studies, more complicated motions and conditions resembling those of an actual patient should be investigated.

## 5 CONCLUSIONS

The dose distributions of gated MRgRT in MRIdian were measured for various target motions using an in‐house‐developed phantom. The effects of the duty cycle, number of beam switches, and target velocity on dose distributions were assessed. The displacement of dose distribution depends on the respiratory condition of a patient and the inherent latency of the treatment machine. The discrepancy in dose distributions when the target was stationary and moving increased with decreasing respiration period, increasing the number of beam switches and target motion velocity. The gating latencies estimated via film measurements using our novel analytical approach agree with those reported in the literature.

## AUTHOR CONTRIBUTIONS

Authors meet all of the following criteria: Substantial contributions to the conception or design of the work; or the acquisition, analysis, or interpretation of data for the work; and drafting the work or revising it critically for important intellectual content; and final approval of the version to be published; and agreement to be accountable for all aspects of the work in ensuring that questions related to the accuracy or integrity of any part of the work are appropriately investigated and resolved.

## CONFLICT OF INTEREST

There is no ethical problem or conflict of interest with regard to this manuscript.
